# "Cough officer screening" improves detection of pulmonary tuberculosis in hospital in-patients

**DOI:** 10.1186/1471-2458-10-238

**Published:** 2010-05-10

**Authors:** Ching-Hsiung Lin, Cheng-Hung Tsai, Chun-Eng Liu, Mei-Li Huang, Shu-Chen Chang, Jen-Ho Wen, Woei-Horng Chai

**Affiliations:** 1 Division of Chest Medicine, Department of Internal Medicine, Changhua Christian Hospital, 135 Nanshiao Road, Changhua, Taiwan; 2 Division of Infectious Disease, Department of Internal Medicine, Changhua Christian Hospital, 135 Nanshiao Road, Changhua, Taiwan; 3 Infection Control Committee, Changhua Christian Hospital, 135 Nanshiao Road, Changhua, Taiwan; 4 Department of Nursing, Changhua Christian Hospital, 135 Nanshiao Road, Changhua, Taiwan

## Abstract

**Background:**

Current tuberculosis (TB) reporting protocols are insufficient to achieve the goals established by the Stop TB partnership. Some countries have recommended implementation of active case finding program. We assessed the effect of Cough Officer Screening (an active screening system) on the rate of TB detection and health care system delays over the course of four years.

**Methods:**

Patients who were hospitalized at the Changhua Christian Hospital (Changhua, Taiwan) were enrolled from September 2004 to July 2006 (Stage I) and August 2006 to August 2008 (Stage II). Stage II was implemented after a Plan-Do-Check-Act (PDCA) cycle analysis indicated that we should exclude ICU and paediatric patients.

**Results:**

In Stage I, our COS system alerted physicians to 19,836 patients, and 7,998 were examined. 184 of these 7,998 patients (2.3%) had TB. Among these 184 patients, 142 (77.2%) were examined for TB before COS alarming and 42 were diagnosed after COS alarming. In Stage II, a total of 11,323 patients were alerted by the COS system. Among them, 6,221 patients were examined by physicians, and 125 of these patients (2.0%) had TB. Among these 125 patients, 113 (90.4%) were examined for TB before COS alarming and 12 were diagnosed after COS alarming. The median time from COS alarm to clinical action was significantly less (p = 0.041) for Stage I (1 day; range: 0-16 days) than for Stage II (2 days; range: 0-10 days).

**Conclusion:**

Our COS system improves detection of TB by reducing the delay from infection to diagnosis. Modifications of scope may be needed to improve cost-effectiveness.

## Background

The 2006 Stop TB partnership, which is advocated by the World Health Organization (WHO), emphasizes expansion of directly-observed treatment short-course (DOTS) as a tuberculosis (TB) control strategy [1]. Passive case finding (PCF), defined as the detection of active TB cases among symptomatic patients who voluntarily present to healthcare facilities, is an important part of DOTS [2]. When local healthcare facilities are functioning efficiently and TB prevalence is low, DOTS may be sufficient. DOTS has had notable success in countries with low prevalence of HIV [3].

However, PCF can lead to delays in the diagnosis and treatment of TB, leading some clinicians and the public health systems of some countries to recommend implementation of active and/or enhanced case finding (ACF, ECF) [4]. ACF and ECF seek to improve early detection and treatment of TB. ACF requires face-to-face contact, onsite evaluations, widespread use of radiography, house-to-house surveys, out-patient case detection, and the monitoring of high risk people who have not reported to healthcare facilities on their own. ECF, which should only be employed with a strong PCF system, is less costly than ACF. ECF uses public education campaigns to increase voluntary screening of target populations. Both strategies aim to detect and treat TB patients earlier than would occur otherwise and to reduce disease transmission [5].

In Taiwan, TB is the most significant notifiable infectious disease, and several hospital outbreaks have been reported in recent years. The incidence of TB has increased from 62 per 100,000 in 1998 to 74 per 100,000 in 2004 and an estimated 15,000 cases have been reported to the national Centre of Disease Control each year since 2002 [6]. Taiwan has acknowledged the importance of the Stop TB initiative, and has had an aggressive TB monitoring system since 1997. This system requires medical personnel to report all suspected and confirmed cases of TB via an electronic TB reporting enquiry system (established in 2001) under a no-report-no-reimbursement policy/notification-fee policy [7]. Under this policy, the National Health Insurance Program rewards healthcare facilities for reporting suspected cases within 7 days prior to treatment, and disentitles reimbursement to facilities that do not report suspected cases.

Recent reports of nosocomial TB outbreaks in Taipei, caused by delays in diagnosis and treatment [8,9], suggest that institutionalized TB reporting and DOTS alone may be insufficient to achieve targets established by Stop TB. In particular, previous studies have shown that TB diagnosis can be very complicated in hospitals, and delayed diagnosis is most likely to occur for hospitalized patients [10]. A recent study showed that TB patients in non-pulmonary/infectious disease wards had longer delays in suspicion, treatment, and respiratory isolation [11].

We have previously described a Cough Officer Screening (COS) program for hospital inpatients [12]. COS is a targeted ACF system in which a computerized physician order-entry system reminds physicians to survey patients who have had coughs for more than 5 days, as recorded by "Cough Officer" nurses. This system is similar to the use of "Ward Cough Officers" in some medical wards of Blantyre, Malawi, which has helped to identify patients with TB symptoms, assisted in collection of sputum, and facilitated delivery of laboratory results [13]. Cough is the most common symptom of active TB [4], and patients with a cough lasting two or more weeks have higher yields of sputum smear-positive TB [14]. WHO's Practical Approach to Lung Health (PAL) employs a symptomatic ACF to improve the case detection component of DOTS for TB control by focussing on patients who have had coughs for 2 to 3 weeks [14].

In the present two-stage study, in which a Plan-Do-Check-Act (PDCA) cycle analysis was implemented after the first stage, we evaluated the effect of a COS program on the rate of TB detection and health care system delays over a period of four years.

## Methods

### Study design

Hospital inpatients who were admitted from September 2004 to July 2006 (Stage I), and August 2006 to August 2008 (Stage II) with various diagnoses to all departments and wards of Changhua Christian Hospital (Changhua, Taiwan) were enrolled. After a Plan-Do-Check-Act analysis at the end of Stage I, we excluded ICU and paediatric patients in Stage II, because a relatively high percentage (21/42, 50%) of all TB cases were in intensive care units, and it was very difficult to monitor coughing in the isolation of an ICU. We also excluded paediatric patients in Stage II, because pulmonary TB is very rare in Taiwanese children, and sputum collection from these patients can be difficult.

This cough officer screening program is recommended by our National Center of Disease Control and approved by our hospital's infection control committee. The study design is retrospective data-analysis and the data are all retrieved from Infectious Control Databank of Changhua Christain Hospital. All the data is decoding and managed in compliance with the Helsinki Declaration and no any patient's personal data were involved.

### Setting of the study

The Changhua Christian Hospital is a non-profit medical center and teaching hospital established in 1896. This facility offers over 60 clinical speciality and sub-speciality departments and has approximately 4,400 inpatient admissions monthly. The average length of stay for inpatients is 7 days. There are 1,413 inpatient beds, with 964 in the acute care ward, 156 in the chronic care ward, 166 in the adult and paediatrics intensive care unit, and 127 in special care wards, such as the respiratory care center and hospice care.

### Cough officer screening protocol

Our COS protocol was devised to allow early detection of pulmonary TB and to prevent its spread within the hospital [12]. The COS recorded all patients' coughs for as long as they were in the hospital. Cough officers (mostly nurses in the general ward) were health care workers who were best able to monitor patient coughing. All cough officers received training in our department of infectious disease control with regard to methods for questioning patients, and for recording cough conditions and duration in our computerized COS system.

When nurses used their computers to check orders every morning during patient admission, they recorded all patients who complained of cough, including during the pre-admission period. If a patient complained of cough at any other time of day, the nurses would also record this on their computers. The computerized physician order entry system reminded doctors to survey patients who had a cough for more than 5 days, so they could perform chest radiography, sputum smears, and cultures for pulmonary TB. The cough duration was the interval from the first day of cough to the day that the doctor prescribed examinations for suspected pulmonary TB. If a patient had a cough for more than 5 consecutive days, an alarm window on the computer screen would remind doctors to schedule chest radiography, sputum smears, or cultures for suspected pulmonary TB each day until such tests were performed. A doctor could ignore the alarm, and give other orders if he ruled out pulmonary TB, if the patient was already being treated for tuberculosis, or if he was a consultant. When a sputum smear or culture tested positive for pulmonary TB, the patient was isolated and given anti-tuberculosis treatment.

### Tuberculosis diagnostic procedures

The physician could prescribe chest radiography, sputum smear/culture, or both for the diagnosis of patients who tested positive in the COS system. The physician might only prescribe sputum smear/culture if the patient had a previous chest X-ray, in which case, three sets of sputum were collected. Regardless of the outcome of chest radiography and sputum smear/culture, the physician might make a diagnosis of TB based on clinical manifestations (symptoms) of the patient.

### Statistical analysis

Categorical data is presented as numbers with percentages and continuous data is presented as medians, with minimums and maximums where appropriate for non-normal distributions. The Wilcoxon rank-sum test was used to assess differences in healthcare system delays between the two stages of COS implementation. For statistical analysis, all assessments were two-sided and evaluated at the 0.05 level of significance. Statistical analyses were performed using SPSS 15.0 statistical software (SPSS Inc, Chicago, IL, USA).

## Results

**Figure 1:**
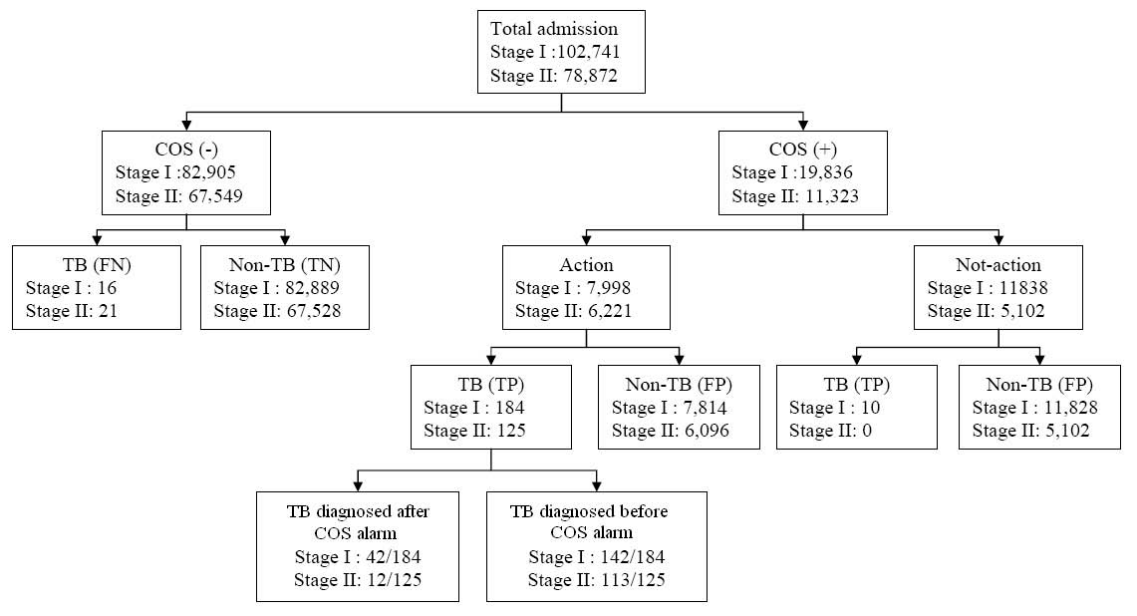
**Flow chart summary of TB detection by cough officer screening (COS) from the time of admission to the time of treatment**. Stage I: September 2004 to July 2006; Stage II: August 2006 to August 2008; TP: true positive; FP: false positive; TN: true negative; FN: false negative.

Figure 1 summarizes the results of our TB detection program from the time of patient admission to initiation of treatment under our cough-officer-screening (COS) system. There were 102,741 patients admitted in Stage I and 78,872 patients in Stage II. Stage II had fewer patients because we implemented a Plan-Do-Check-Act (P-D-C-A) analysis to exclude patients admitted to the ICU and paediatrics departments after completion of Stage I. By excluding the TB patients in the ICU, we found that 81% and 100% of TB patients in the Internal Medicine Department in Stage I and Stage II, respectively. This suggests that the majority of COS (+) patients were in this department.

The COS system identified 19,836 from 102,741 patients (19%) with alarms in Stage I,. Physicians examined 7,998 of these 19,836 patients (40%). A total of 184 of these 7,998 patients (2.3%) were diagnosed with TB. However, doctors ordered TB examinations for 142 of these 184 patients (77.2%) before COS alarming. Thus, 42 of 184 patients (23%) were diagnosed with TB only after physicians were alarmed by the COS system. These patients probably would have remained undiagnosed for a period of time if our COS program had not been implemented.

The COS system identified 11,323 of 78,872 patients (14%) with alarms in Stage II. Physicians examined 6,221 of these 11,323 patients (55%). A total of 125 of these 6,221 patients (2.0%) were diagnosed with TB. However, doctors ordered TB examinations for 113 of these 125 patients (90%) before COS alarming. Thus, 12 of 125 patients (9.6%) were diagnosed with TB only after physicians were alarmed by the COS system. Again, these patients probably would have remained undiagnosed for a period of time if our COS program had not been implemented.

**Figure 2:**
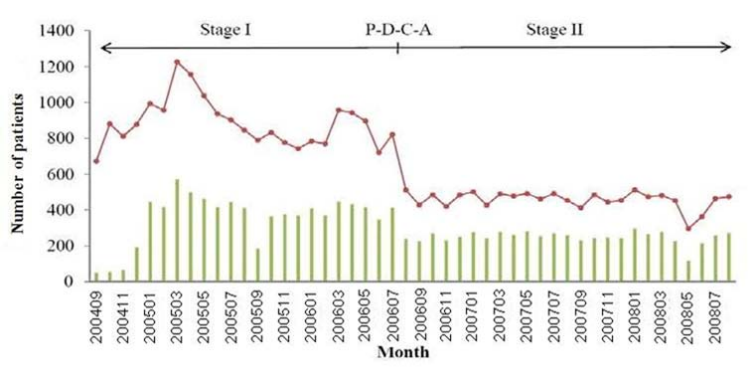
**COS alarm frequency and number of diagnostic procedures undertaken during Stage I and Stage II**. Red points indicate the number of cases that elicited an alarm; green bars indicate the number of diagnostic procedures (chest X-ray or sputum examination) that were taken.

Figure 2 shows the number of COS alarms (red points) and diagnostic procedures undertaken (chest X-ray or sputum examination; green bars) for each month of Stage I and Stage II. This figure shows that there were fewer alarms during Stage II, but that the number of diagnostic procedures undertaken did not decrease. In fact, the mean percentage of actions taken by physicians following alarm was 39.66% (Range: 6.25%-52.17%) in Stage I and 54.33% (Range: 39.39%-58.95%) in Stage II. This indicates that the doctors were more aware of the critical role of COS in TB prevention during Stage II, so that more patients had alarms and examinations.

Table 1 shows the sensitivity, specificity, positive predictive value (PPV), and negative predictive value (NPV) of our COS system. A definite diagnosis (true positive; TP) of TB was defined as a positive Mycobacterial culture. Our COS had similar and relatively high sensitivity and specificity in Stages I and II, with nearly 100% NPV, but very low PPV (~1%).

**Table 1:** Sensitivity, specificity, positive predictive value, and negative predictive value of the COS system, in which diagnosis was based on a positive culture.

	Sensitivity (= TP/(TP + FN))	Specificity (= TN/(FP + TN))	PPV (= TP/(TP + FP))	NPV (= TN/(TN + FN))
Stage I	92.38%	80.84%	0.98%	99.98%
Stage II	85.62%	85.78%	1.10%	99.97%

TP: true positive; FN: false negative; TN: true negative; FP: false positive; PPV: Positive predictive value; NPV: Negative predictive value.

Table 2 summarizes the length of time from admission to alarm, alarm to diagnostic action, admission to diagnosis, and diagnosis to treatment via the COS alarm system. There were 42 patients (17 in the internal medicine department, 4 in the surgical department, and 21 in the ICU) in stage I, and 12 patients (all in the internal medicine department) in stage II. The times from admission to alarm, admission to diagnosis, and diagnosis to treatment were similar in Stage I and Stage II. However, the median time from alarm to action was significantly less during Stage I than Stage II (P < 0.05).

**Table 2:** Length of time from admission to alarm, alarm to diagnostic action, admission to diagnosis, and diagnosis to treatment via the COS alarm system.

Duration^a ^(From→To)	Stage I (42 patients)	Stage II^b ^(12 patients)	P-value^c^
Admission → Alarm	5(0,20) days	2(0,14) days	0.255
Alarm → Diagnostic action	1(0,16) days	2(0, 10) days	0.041*
Admission → Diagnosis	18(2,163) days	14(4,55) days	0.435
Diagnosis → Treatment	0(0,7) days	0(0,2) days	0.934

^a ^Duration expressed as median (minimum, maximum) days.

^b ^The duration from admission to alarm in Stage II was less than 5 days because some patients self-reported coughing prior to admission.

^C ^p-value determined by non-parametric Wilcoxon rank-sum test.

* p-value less than 0.05

Table 3 presents the demographics of patients who had confirmed pulmonary TB. These patients had a median age of 76.0 during Stage I and 75.5 during Stage II, median cough duration of 7.0 days during for Stage I and 8.0 days during Stage II, and median time from alarm to diagnosis of 14 days for stage I and 12.5 days for stage II. For both stages, 50% of TB patients initially had negative sputum smears, but eventually tested positive.

**Table 3:** Demographic data of patients with confirmed TB.

Demographics*	Stage I (N = 42)	Stage II (n = 12)	p-value
Age, years	76.0 (64.0,81.3)	75.5 (70.0,85.0)	0.312
Gender, Male (%)	28 (66.7%)	9 (75.0%)	0.732
Duration of cough, days	7.0 (5.0,7.25)	8.0 (7.0,9.0)	0.038^†^
From alarm to diagnosis, days	14 (3, 26)	12.5 (5.0,19.0)	0.950
Smear-culture result			
Smear negative, culture positive	21 (50%)	6 (50%)	1.000
Smear positive, culture positive	21 (50%)	6 (50%)	

* Demographics were summarized as median (Q1,Q3) for age, duration of cough, time from alarm to diagnosis with non-parametric Wilcoxon rank-sum test, and as n (%) for gender, outcome of smears and cultures with Chi-square/or Fishers' exact test.

^† ^p-value < 0.05 indicated the duration of cough (days) was significantly higher in stage II than stage I.

## Discussion

The results of this study of our COS program indicate that the delay of the healthcare system in responding to TB depends on the population of patients who are surveyed (Figure 2). Among patients with alarms who were subsequently examined by sputum smear and bacterial culture, only 2.3% (Stage I) and 2.0% (Stage II) were diagnosed with TB. This suggests that the population of patients being surveyed by our COS system may need revision. Alternatively, the cut-off point (cough for more than 5 days) may need to be extended to reduce the number of false alarms.

The results reported here are similar to those of our previous study [12], but very different from those of Banda et al. [15]. Banda et al. reported a 35% TB detection rate among the 180 patients referred from a general outpatient department to a "chronic cough room" at a hospital in Blantyre, Malawi. The high detection rate in this report may be due to the use of more refined criteria for suspicion of TB, higher prevalence of TB in this population, and the poorer quality of healthcare and diagnostic facilities. All of the patients in the Malawi study were more than 15 years-old, coughed for more than one week but less than 3 weeks, were refractory to short-course antibiotics (self-administered or administered by outpatient staff), and had no previous history of TB.

The WHO's PAL program recommends using a cough duration of 2 to 3 weeks for diagnostic evaluation of TB [16]. In Europe, 36 of 50 countries (72%) recommend sputum examination of patients who have coughs that last more than 3 weeks [17]. A study of TB in India recommended diagnostic evaluation of patients who have coughs that last more than 2 weeks [14]. A study of TB in Cuba recommended the use of an ACF that included patients who coughed 3 weeks or more [18]. Researchers of TB among Canadian Plains Aborigines argued that diagnostic procedures be initiated for patients who cough for more than 1 month and have unexplained fever for more than 1 week [19]. Thus, as suggested by the study of den Boon et al. [4], use of less stringent criteria for initiation of diagnostic procedures (e.g. 5 days of coughing) may lead to a high rate of false positives, but also leads to earlier identification of TB-positive patients, thereby allowing for earlier treatment and reduced rate of transmission.

We analyzed the sensitivity, specificity, positive predictive value (PPV), and negative predictive value (NPV) of our COS system. COS had a relatively high sensitivity and specificity in both stages, suggesting that it is an effective system for early screening of hospitalized TB patients. Both stages of our study had almost 100% NPV, but very low (~1%) PPV. This suggests that very few COS (-) patients were ultimately diagnosed as having TB, but only ~1% of COS (+) patients were eventually diagnosed as having TB (based on Mycobacterial cultures). Acute cough is a complication of many diseases [20], so our COS system would be expected to produce many false positives. Clearly, patients with acute cough should be considered as possibly having TB, but not to the exclusion of other common diseases. Our finding is consistent with a previous WHO study in which TB was diagnosed in only 1.5% of patients in Morocco who had respiratory problems (such as persistent cough) and were initially suspected of having TB [14]. Another study found that 77% of patients initially diagnosed as having TB were TB smear-positive [21].

Among the patients that we diagnosed as having TB, 77.2% were diagnosed with TB before a COS alarm during Stage I. The physicians apparently suspected TB based on their initial clinical examinations. However, in Stage II, doctors ordered TB examinations for 90% of TB patients before a COS alarm. This difference might due to the increased awareness of TB in Stage II (Figure 2), or because of interference of TB diagnosis by other severe symptoms among patients in the ICU during Stage I. In fact, our COS system identified 42 patients (22.8% of total TB patients) in Stage I and 12 patients (9.6% of total TB patients) in Stage II with TB. Among those 54 patients, 50% of patients initially had negative sputum smears, but eventually tested positive (Table 3). Without our COS, physicians may have ignored these patients, and they could have become sources of nosocomial infections in our hospital. Thus, although it was not an objective of this study, our COS system appeared to reduce the nosocomial transmission of *M. tuberculosis*.

Exclusion of ICU and paediatric patients in Stage II did not result in a significant change in health care system delay (Table 1). In fact, there was a modest increase in time from COS alarm to diagnostic action in Stage II (Stage I: 1(0, 16) days; Stage II: 2(0, 10) days; p = 0.041). This may be due to the reduced concern about nosocomial cross-infection outside the ICU. In addition, treatment delay may be less in Stage I patients with life-threatening conditions if they had a short history of cough in the ICU. These results suggest that alternative criteria should be considered for screening ICU patients.

The average time from admission to diagnosis was 24 days for Stage I and 19 days for II (data not shown). This points to another advantage of COS system: it can identify patients infected by *M. tuberculosis *during the entire admission period. We suggest that a well-implemented COS program should be able to reduce or even prevent nosocomial transmission of TB.

This study has left some important questions unanswered. First, the optimum cough duration that should be used for the suspicion of pulmonary TB remains unknown, and may in fact differ for different populations of patients. Second, there are groups known to be at high-risk for development of pulmonary TB. If we had limited our COS protocol to these high-risk patients, the sensitivity of our COS might have been better. Clearly, this requires further investigation. Finally, our COS system resulted in high rates of examination, but low rates of diagnosis. This requires cost effectiveness analysis of the COS system in future study.

In association with physicians' clinical diagnoses, COS appears to improve detection of TB. However, modifications of the scope of our COS may be needed to improve the efficacy. Approximately 10-20% of TB patients may be missed if a COS system is not implemented. Implementation of a COS system may also encourage doctors to be more aware of the critical role of cough in identification of TB.

## Conclusions

TB has been a significant public health problem in Taiwan for many years, with an annual incidence greater than 70 per 100,000, and significantly higher incidence in the rural mountainous regions [6,22]. We suggest that other Taiwanese hospitals and health care centers consider implementation of a COS system initially in their Internal Medicine Departments, because we found that most COS (+) patients were in this department. In addition, we recommend that the cough duration for COS system should be optimized and the cost effect of the COS system should be analyzed before implementation.

## Competing interests

The authors declare that they have no competing interests.

## Authors' contributions

C-HL, C-HT, and C-EL participated in the design of the study and performed the statistical analysis. M-LH and S-CC conceived the study, and participated in its design and coordination. C-HL, J-HW, and W-HC helped to draft the manuscript. All authors read and approved the final manuscript.

## Pre-publication history

The pre-publication history for this paper can be accessed here:

http://www.biomedcentral.com/1471-2458/10/238/prepub
